# External Pressures or Internal Governance – What Determines the Extent of Corporate Responses to Climate Change?

**DOI:** 10.1002/csr.1473

**Published:** 2017-12-19

**Authors:** Matthias Damert, Rupert J. Baumgartner

**Affiliations:** ^1^ Institute of Systems Sciences, Innovation and Sustainability Research and FWF‐DK Climate Change University of Graz Graz Austria

**Keywords:** climate change, corporate action, automotive industry, institutional environment, corporate governance, stakeholder

## Abstract

To prevent adverse effects from climate change, it is vital to involve the private sector in mitigation efforts. So far, however, research has insufficiently addressed the determinants of corporate action in specific industries. Our paper aims at bridging this gap by empirically analyzing the global automotive industry's response to climate change mitigation issues. We use publicly available information from 105 sector leaders to investigate the role of external institutional pressures and intra‐organizational governance in the extent of corporate action. Based on a multiple regression analysis, we find that organizational involvement and the integration of climate change into risk management exhibit the greatest influence. Moreover, companies with business activities that necessitate interaction with the end consumer tend to be most active. Our analysis furthermore indicates that neither the stringency of a firm's home country's climate policy regime nor the degree of internationalization is associated with a higher implementation level of response strategies. © 2017 The Authors. *Corporate Social Responsibility and Environmental Management* published by ERP Environment and John Wiley & Sons Ltd.

## Introduction

In November 2016, the Paris agreement entered into force with the aim of limiting global warming to a maximum of 2^o^ Celsius (United Nations Framework Convention on Climate Change, 2017). It is expected that more and more countries will implement stringent policies calling for ambitious involvement of the private sector in the near future (International Finance Corporation, 2016). This reduces business risks since regulatory uncertainty diminishes, but it also creates new challenges associated with compliance, changing consumer attitudes, and pressures from stakeholders and shareholders to reduce greenhouse gas (GHG) emissions (Wittneben *et al*., 2012).

Management research has only rather recently taken up the topic of climate change, as it is often treated as being integral to corporate environmental management. However, it deserves special attention because of its global dimension and the urgency of necessary action (Luo and Tang, 2016). So far, most studies have looked at the characteristics of business responses to climate change, both theoretically (Okereke *et al*., 2012) and empirically (Kolk and Pinkse, 2005; Lee S‐Y, 2012; Weinhofer and Hoffmann, 2010). While initially scientific focus was on developed countries (Levy and Kolk, 2002), emerging and developing countries recently received an increasing amount of attention (Amran *et al*., 2016; Jeswani *et al*., 2008). Another stream of literature concentrates on analyzing factors that determine corporate climate change strategies. Internal factors that have been considered include, inter alia, management characteristics (Ciocirlan and Pettersson, 2012), business motivations (Boiral *et al*., 2012), and financial performance (Stanny and Ely, 2008). Also external pressures from regulation, shareholders, stakeholders, and customers have been found to be determinants of corporate responses (Böttcher and Müller, 2015; Sprengel and Busch, 2011; Jira and Toffel, 2013). Apart from that, sector affiliation seems to play a crucial role in shaping climate strategies (Okereke and Russel, 2010).

Notwithstanding the importance of existing research, the majority of studies on determinants of corporate climate action are based on cross‐sectoral analyses and thereby neglect intra‐sectoral dynamics (Weinhofer and Hoffmann, 2010; Glienke and Guenther, 2016). In other words, the importance of internal and external factors for triggering business responses might vary across sectors. Although most cross‐industry analyses have taken sector affiliation into account and revealed significant differences (Boiral *et al*., 2012; Luo and Tang, 2016), there is little empirical evidence for the underlying causal mechanisms. Moreover, most research that analyzes intra‐sector variation in strategies employs qualitative methods leading to a limited generalizability of findings (Levy and Kolk, 2002; Okereke and Russel, 2010; Busch and Schwarzkopf, 2013). In consequence, the question of why corporate approaches to climate change mitigation issues differ remains insufficiently answered (Luo and Tang, 2016).

To bridge this research gap, the research objective of the present study is to develop a better understanding of the importance of internal and external factors for triggering corporate responses to climate change within a particular industry. In terms of external pressures to act on climate change, our paper focuses on examining the role of (1) the stringency of climate policies in a company's home country; (2) the degree of a company's exposure to foreign institutional environments; and (3) the company's position in the supply chain (i.e. its closeness to the end consumer). We also consider two important intra‐organizational factors related to corporate governance, namely, a company's risk management approach and the level of organizational involvement regarding climate change issues.

Our study makes three important contributions to the literature on business responses to climate change. First, we help disentangle the causal mechanisms that underlie corporate action on climate change within an industry. Second, by testing propositions from institutional and stakeholder theories, we provide empirical evidence for the effectiveness of distinct regulatory environments and stakeholder pressures. Third, by taking a resource‐based view (RBV) perspective, we generate knowledge about the importance of organizational resources and corporate governance for the implementation of climate change strategies.

The remainder of this paper is organized as follows. First, we review the literature on business responses to climate change to derive research hypotheses. We subsequently depict the role of the global automotive industry for combating climate change and explain why we have chosen it as the object of investigation. We then empirically examine the relationship between internal and external factors and the extent of corporate climate action through a regression analysis based on a sample of 105 leading automobile manufacturers and suppliers.

## Theoretical Background and Research Hypotheses

Business responses to climate change mitigation issues can be classified into biophysical and politico‐economic mitigation (Okereke *et al*., 2012). While the former approach is geared towards reducing a firm's environmental impact through reducing GHG emissions, the latter aims at managing socio‐economic business risks attached to climate change mitigation. So far, most scholars have concentrated on biophysical mitigation measures, such as the improvement of existing, or the development of new, products and production processes (Kolk and Pinkse, 2005; Cadez and Czerny, 2016), low‐carbon logistics (Böttcher and Müller, 2015), the acquisition of emission credits through trading schemes or offsetting mechanisms (Weinhofer and Hoffmann, 2010), or the involvement of suppliers in emission reduction efforts (Lee S‐Y, 2012). Primarily focusing on these aspects is understandable since the reduction of emissions is at the centre of the climate change mitigation debate. Yet, it fails to capture the socio‐political facets of business responses to climate change, such as lobbying (Levy and Kolk, 2002), carbon disclosure (Freedman and Jaggi, 2009), and stakeholder cooperation (Jeswani *et al*., 2008; Sprengel and Busch, 2011). The literature on corporate climate change strategies remains largely divided and only few studies adopt a holistic perspective (Lee S‐Y, 2012). To overcome these shortcomings, we consider both biophysical and politico‐economic activities in our study. In line with Yunus *et al*. (2016), we therefore understand corporate action in the context of climate change as ‘carbon measurement, reporting, reduction, trading and other measures to mitigate climate change‐related risks, seize opportunities and enhance corporate competitiveness in a carbon‐constrained market place.’ page 158.

### External Drivers for Corporate Climate Change Action

Institutional theory suggests that a company's behaviour is shaped by three types of external institutional forces: (1) normative (i.e. the need for complying with social and cultural norms); (2) coercive (i.e. formal rules established by regulations); and (3) mimetic (i.e. the tendency to imitate competitors' behaviour to avoid uncertainty) (DiMaggio and Powell, 1983). In the case of climate change, coercive pressures in the form of strict regulations may not only directly affect companies' behaviour but also lead to greater societal concerns over corporate compliance and increased stakeholder scrutiny (Lorenzoni and Pidgeon, 2006). The introduction of climate policies has forced companies to revise their business practices to reach regulatory compliance (Pinkse, 2007; Pinkse and Kolk, 2009). In line with stakeholder theory, policies can also amplify pressure exercised by investors, non‐governmental organizations (NGOs), and customers (Jones and Levy, 2007; Martin and Rice, 2010), which is important for the development of corporate responses to climate change (Reid and Toffel, 2009; Pinkse and Busch, 2013). Most studies provide empirical evidence for the influence of different institutional factors on corporate responses to climate change (Luo and Tang, 2016; Cadez and Czerny, 2016). Researchers have outlined the positive relationship between regulatory pressure and the extent of corporate initiatives geared towards climate change mitigation (Boiral *et al*., 2012; Böttcher and Müller, 2015). The legal framework of a company's home country especially is of great importance, resulting in a ‘home country effect’ (Levy and Kolk, 2002; Weinhofer and Hoffmann, 2010; Amran *et al*., 2016). We therefore propose:

*A more stringent climate policy regime in a company's home country is positively related to the level of corporate action on climate change*.

Institutional theory furthermore assumes that in a particular organizational field corporate behaviour converges in the long run due to similar institutional pressures, which is called institutional isomorphism (DiMaggio and Powell, 1983). Busch and Schwarzkopf (2013), for example, find little variation in strategies among large car manufacturers from different countries. This supports the notion of institutional isomorphism, but it also questions the importance of the home country effect. If multinational companies with different regional affiliations pursue similar strategies, the institutional settings of their foreign sales markets might be more influential than home country factors. This has been termed the ‘host country effect’ (Amran *et al*., 2016). As climate change is a global issue, multinational companies are exposed to various kinds of regulations and stakeholders. Foreign markets sometimes possess stricter environmental laws and standards. Therefore, to maintain their licence to operate, companies are forced to adapt their business strategy accordingly (Chakrabarty and Wang, 2013). Respective companies also tend to be more experienced in implementing appropriate response measures (Bansal, 2005). Hence, we hypothesize:

*More globalized companies exhibit a higher level of climate action*.

The environmental management literature proposes another external factor that triggers corporate action on climate change: closeness to the consumer, which is a feature of companies ‘that supply goods or services directly into consumer markets instead of supplying to another business entity’ page 141 (Haddock‐Fraser and Fraser, 2008). While suppliers predominantly conduct business‐to‐business (B2B) activities, original equipment manufacturers (OEMs) focus on activities that also involve interaction with the end consumer of their products. OEMs may therefore be susceptible to a higher degree of consumer pressure (Bansal and Roth, 2000). Kolk and Pinkse (2007) add that ‘the position of the company in the supply chain determines to which extent a company depends on its customers and thus follows a concomitant climate strategy’ page 374. Empirical studies have found a positive link between a company's closeness to the end consumer (*C2C*) and both environmental disclosure (Haddock‐Fraser and Fraser, 2008) and environmental proactivity (Haddock‐Fraser and Tourelle, 2010). On this basis, we propose:

*Companies with business activities that are closer to the end consumer exhibit a higher level of climate action*.

### Intra‐Organizational Determinants of Corporate Climate Change Action

From a theoretical point of view, corporate governance in the context of climate change is connected to the (natural) RBV of a firm (Hart, 1995; Backman *et al*., 2017). The RBV suggests that investing in specific organizational resources can trigger environmental action and lead to competitive advantages (Barney, 1991). Among these resources, pronounced managerial skills and employees' awareness of environmental issues represent important drivers for action of corporations on climate change (González‐González and Zamora‐Ramírez, 2013; Amran *et al*., 2016). Moreover, assigning responsibilities for climate change mitigation to senior managers and introducing outcome‐based remuneration schemes provide incentives for implementing appropriate response strategies, including emission reduction activities, stakeholder initiatives, and the exertion of influence on climate politics (Kolk and Levy, 2001; Okereke *et al*., 2012; Backman et al., 2017). Hence, the following research hypotheses are derived:

*Companies that have assigned climate change responsibilities to managers or committees exhibit a higher level of climate action*.

*The presence of a performance‐based remuneration scheme is associated with a higher level of climate action*.

*Companies with employees who are aware of climate change issues exhibit a higher level of climate action*.

Another important aspect of corporate governance is the management of risks. Companies face various challenges in the context of climate change mitigation, such as carbon taxes, mandatory emission trading schemes, or changing consumer perceptions (Hoffman, 2006; Busch and Hoffmann, 2007; Galbreath, 2010). A growing demand for climate‐friendly products, for example, potentially leads to competitive disadvantages if such products are not part of a firm's portfolio. Insufficient action on climate change can also cause reputational, financial, and litigation risks (Busch and Hoffmann, 2007). Climate change risks should hence be integrated into corporate risk management and addressed by appropriate risk mitigation strategies (Weinhofer and Busch, 2013). To our knowledge, empirical research about the impact of corporate risk management approaches on climate change strategies is still lacking. However, a simulation study by Bleda and Shackley (2008) based on organizational behaviour theories suggests that a company's awareness of climate change risks can prompt the implementation of measures aimed at enhancing an organization's adaptive capacity. We thus propose:

*The integration of climate change into corporate risk management is associated with a higher level of climate action*.

## Climate Change Mitigation and the Automotive Industry

In 2011, the transportation sector's share in global carbon dioxide (CO_2_) emissions amounted to 22% [Intergovernmental Panel on Climate Change (IPCC), 2014]. Road transport is the sector's main source of GHG emissions and exhibited an immense 52% growth rate between 1990 and 2011, especially in developing countries (International Energy Agency, 2013; IPCC, 2014). Although the efficiency of vehicles has improved over the years, emissions are expected to grow even further in the next decades (IPCC, 2014). On account of these developments, regulatory bodies have implemented a variety of policy measures to reduce mobility‐related GHG emissions. On the one hand, the introduction of carbon taxes on cars, congestion charges, investments in public transportation, and the promotion of car‐sharing services incentivize behavioural changes on the part of consumers (IPCC, 2014). On the other hand, restrictive fleet emission limits, fuel economy standards, and subsidies for alternative propulsion systems, such as electric vehicles, are meant to facilitate the development of technological solutions for emission reductions [International Council on Clean Transportation (ICCT), 2014]. In this context, the automotive industry increasingly faces risks with regard to regulatory compliance and reputation. As the recent Volkswagen scandal has shown, failing to comply with emission standards can have serious negative financial and reputational consequences for affected companies (Cremer, 2016). Yet, as the scandal caused a shift in Volkswagen's strategy towards the accelerated development of low‐carbon vehicles, it also demonstrates the effectiveness of institutional pressure for corporate action on climate change.

Although it is important to look at the industry's indirect carbon footprint caused by the use of its products, the direct impact of its business activities should not be neglected. While OEMs' direct emissions are dwarfed by the CO_2_ emitted during the use phase of cars, suppliers' operations often involve highly energy‐intensive manufacturing processes such as the production of steel, aluminium, plastic, or glass components (Böttcher and Müller, 2015). Moreover, extensive outsourcing practices by OEMs have led to a situation in which up to 70% of value creation and a large share of innovation are carried out by suppliers (Di Botonto, 2014). This emphasizes the immense role of automotive suppliers in reducing the industry's carbon footprint. It also highlights how the effectiveness of manufacturers' climate change action depends on the performance of their suppliers (Lee K‐H, 2012). However, most research has focused on either auto manufacturers (Kolk and Levy, 2003; Busch and Schwarzkopf, 2013) or companies further up the supply chain (Böttcher and Müller, 2015; Jira and Toffel, 2013). To fill this gap, our study considers both types of companies.

## Research Design

### Data Collection and Sample

Our empirical analysis relied on cross‐sectional secondary data from leading automotive manufacturers and suppliers. Initially, we identified the largest OEMs and suppliers based on annual sales volume figures obtained from industry databases. To account for the global nature of the industry, we did not limit our sample to specific regions. We then searched for publicly available information, including corporate social responsibility, sustainability, environmental, and annual reports, company websites, codes of conduct, and press releases. Because dates for fiscal years vary depending on corporate accounting standards, we collected documents that cover company activities in both 2013 and 2014. We also sourced information from the CDP (formerly the Carbon Disclosure Project), Thomson Reuters' Worldscope and Asset4 databases, and websites of trade associations and business initiatives, such as the United Nations Global Compact and the World Business Council on Sustainable Development. The final sample consisted of 105 companies that provided sufficient data for the purpose of our study. Table 1 shows the composition of our sample in terms of supply chain position and regional affiliation. When looking at the sample, there seems to be a bias towards Japanese companies, which make up 40% of the total sample. This figure, however, is in line with official statistics from the industry association Organisation Internationale des Constructeurs d'Automobiles (2014) and the industry magazine *Automotive News* (2014) showing that 40% of the top ten OEMs and about one‐third of the top 100 suppliers, respectively, are headquartered in Japan.

**Table 1:** Supply chain position and home country of companies in the sample

Countries	Supply chain position		Total
	Suppliers	OEMs	
Austria	1	0	1
Canada	2	0	2
Finland	2	0	2
France	5	2	7
Germany	8	5	13
India	3	2	5
Italy	1	1	2
Japan	32	10	42
Netherlands	0	2	2
Norway	1	0	1
South Africa	1	0	1
South Korea	3	2	5
Spain	1	0	1
Sweden	1	3	4
Switzerland	1	0	1
Turkey	1	1	2
UK	1	1	2
USA	9	3	12
Total	73	32	105

*Note:* OEMs as given in the text.

### Measurement of the Dependent Variable

For determining the extent of corporate climate action, we relied on frameworks proposed in previous studies on business responses to climate change. Specifically, we considered nine types of activities related to biophysical and politico‐economic mitigation (see Table 2). The assessment of the implementation level of these activities was carried out in three steps. First, we conducted a qualitative content analysis of the collected company documents. We extracted information about each of the nine activities based on a code book containing keywords, synonyms, and descriptions, as recommended by Neuendorf (2002). Second, we developed a measurement scheme for the implementation level of activities. For this, we built on approaches used in similar studies (Kolk and Pinkse, 2005; Weinhofer and Hoffmann, 2010; Lee S‐Y, 2012) (see Appendix A). By applying the measurement scheme to the qualitative information extracted from company documents, we assigned a value to each company for each of the nine activities, with values ranging from 1 = ‘not implemented’ to 4 = ‘high implementation level’ (see Table 2 and Appendix A). Two researchers independently went through the rating procedure with an acceptable reliability of 87.9% (percentage of agreement) and a Krippendorff's alpha of 0.72 (Krippendorff, 2013). Lastly, we obtained the extent of corporate action on climate change by summing up the values of all nine activities, expressed by the variable *Climate action*.

**Table 2:** Assessment of climate change activities for determining the extent of corporate action (see also Appendix A)

Activity	Description	Measurement
GHG reduction policy	Setting up GHG inventories and emission reduction targets (Lee S‐Y, 2012)	Numerical values were assigned based on the implementation level of each activity:1 = not implemented 2 = low 3 = medium 4 = high
Product improvements	Improving the carbon footprint of products (Yunus *et al*., 2016)	
Process improvements	Optimizing logistics, plant retrofit, energy‐efficient equipment, and renewable energy sourcing (Böttcher and Müller, 2015)	
New markets and products	Developing low‐carbon products or entering markets in which low‐carbon aspects are a unique selling proposition (Kolk and Pinkse, 2005)	
Supplier involvement	Asking suppliers to reduce emissions and assisting them in implementation (Lee K‐H, 2012)	
Emission compensation	Buying emission credits through ETS or offsetting schemes (CDM, JI) (Weinhofer and Hoffmann, 2010)	
Stakeholder cooperation	Cooperating with companies (e.g. World Business Council on Sustainable Development), politicians (e.g. UN Global Compact), NGOs, and other stakeholders (Kolk and Pinkse, 2007; Sprengel and Busch, 2011)	
Carbon disclosure	Disclosing climate change‐related information (Freedman and Jaggi, 2009)	
Political lobbying	Influencing policymakers through lobbying, funding of research, or voluntary reduction commitments (Eberlein and Matten, 2009; Kolk and Pinkse, 2007)	

*Note:* GHG and NGOs as given in the text. ETS = emission trading scheme, CDM = Clean Development Mechanism and JI = Joint Implementation.

### Measurement of Independent Variables

Concerning external pressures, we used the Climate Change Performance Index (CCPI) for 2013, jointly developed by the NGO Germanwatch and Climate Action Network Europe (Burck *et al*., 2014), for determining the pressure exercised by a company's home country's regulatory regime. Specifically, we constructed the variable *Regulation* for each country in our sample based on the index score for its climate policy, ‘reflecting efforts towards an efficient and low‐carbon society’ page 20 (Burck *et al*., 2014). To approximate a company's exposure to foreign institutional environments, we created the variable *Internationalization* through calculating the ratio of foreign sales to total sales as proposed by Chakrabarty and Wang (2013). Furthermore, we used a dichotomous variable to account for a company's closeness to the end consumer *(C2C)*. We assumed that automobile manufacturers operate much closer to the end consumer compared to suppliers, since the latter mainly engage in B2B activities. Therefore, suppliers were coded with ‘0’ and OEMs with ‘1’.

For measuring internal governance, we created four dummy variables. Regarding organizational involvement, we assigned a value of ‘1’ if the following measures were implemented by the company: (1) assigning responsibilities for climate change to senior managers or dedicated committees (*Management responsibility*); (2) providing financial incentives for achieving corporate emission reduction targets (e.g. through variable remuneration) (*Performance incentives*); and (3) raising employees' awareness of climate change (*Employee awareness*). Regarding *Risk management*, we coded the variable with ‘1’ if climate change was integrated into corporate risk management.

### Control Variables

We used three control variables being frequently adduced to explain variation in corporate environmental action: company size, financial performance, and financial leverage (e.g. Bansal, 2005; Delmas *et al*., 2016; Yunus *et al*., 2016). Larger firms tend to show more extensive action on climate change because of easier access to financial and human resources and a higher public visibility leading to greater societal pressures (Bansal, 2005; Lee S‐Y, 2012). We measured company size (*Size*) by the value of total assets in US million dollars. To ensure normal distribution of the data, we computed the natural logarithm. Financial performance is closely related to the financial health of a company. More profitable companies usually possess more available capital that can be invested in activities in response to climate change (Hart, 1995; Luo and Tang, 2016). We measured financial performance by return on assets (*ROA*). Lastly, we included financial leverage (*Leverage*) measured by the ratio of total debt to total assets because ‘the more a firm depends on funding from creditors, the more likely the firm is to address creditors' expectations regarding climate change and related carbon emission issues’ page 166 (Yunus *et al*., 2016), and to engage in political lobbying (Delmas *et al*., 2016).

### Regression Model

To test our research hypotheses statistically, we selected Equation (1) for the multiple regression:

(1) $$\text{Climate action}=\alpha +\beta_{1}\text{Regulation}+\beta_{2}\text{Internationalization}+\beta_{3}C2C+\beta_{4}\text{Management responsibility}+\beta_{5}\text{Performance incentives}+\beta_{6}\text{Employee awareness}+\beta_{7}\text{Risk Management}+\beta_{8}\text{Size}+\beta_{9}\mathrm{ROA}+\beta_{10}\text{Leverage}+\varepsilon$$

The model was estimated using the ordinary least‐squares (OLS) linear regression method. It should be noted that, consistent with prior research, the financial control variables *ROA* and *Leverage* were lagged one year behind the dependent variable (Luo and Tang, 2016).

## Results

### Descriptive Statistics and Univariate Analysis

Table 3 displays the minimum, maximum, and mean values and the standard deviation of the numerical variables. On average, the level of corporate climate change action is medium (2.89). The descriptive statistics show that the analyzed companies mostly sell their products to foreign markets, as the average percentage of foreign sales amounts to 60%. Also, since *ROA* and *Leverage* figures reach 4.88 and 0.63, respectively, the companies in our sample can be described as predominantly financially healthy.

**Table 3:** Descriptive statistics

Variables	Minimum	Maximum	Mean	SD
Climate action	1.33	4.00	2.89	0.59
Regulation	0.01	0.15	0.08	0.04
Internationalization	0.05	0.99	0.60	0.23
Size	5.39	8.64	6.98	0.67
ROA	‐5.69	13.90	4.88	3.79
Leverage	0.19	0.91	0.63	0.15

*Note:* ROA as given in the text.

Table 4 reports the bivariate Pearson correlations between the variables. As expected, most of the independent variables are significantly and positively correlated with *Climate action*. Surprisingly, neither *Regulation* nor the financial performance indicators are significant predictors. The internal governance variables, however, mostly show a moderate correlation with *Climate action*. *Size* is significantly correlated with both *Climate action* and *C2C*. These results seem reasonable, as compared to their suppliers OEMs are usually larger in size and therefore also possess more organizational resources to implement climate change measures.

**Table 4:** Pearson correlations

Variables	1	2	3	4	5	6	7	8	9	10	11
1 Climate action	1										
2 Regulation	0.094	1									
3 Internationalization	0.212*	0.314**	1								
4 C2C	0.522**	0.128	0.106	1							
5 Management responsibility	0.438**	−0.070	−0.033	0.203*	1						
6 Performance incentives	0.618**	−0.021	.0.128	0.276**	0.231*	1					
7 Employee awareness	0.313**	−0.079	0.116	0.159	0.006	0.258**	1				
8 Risk management	0.617**	0.192	0.262**	0.283**	0.241*	0.587**	0.159	1			
9 Size	0.690**	0.097	0.235*	0.574**	0.186	0.544**	0.250*	0.466**	1		
10 ROA	0.032	0.273**	−0.093	−0.044	−0.022	0.030	−0.025	0.072	−0.096	1	
11 Leverage	0.083	0.061	0.068	0.309**	−0.012	0.070	0.000	0.135	0.169	−0.371**	1

*Note: n* = 105;

** *p* < 0.01;

* *p* < 0.05. C2C and ROA as given in the text.

### Multivariate Analysis

Before estimating the proposed statistical model, we tested our data concerning the underlying assumptions of multiple regression. To ensure the normality of independent variables, we inspected histograms and values for kurtosis and skewness. Apart from *Size*, none of the variables showed significant deviations from normal distribution. We therefore transformed *Size* by computing the natural logarithm to ensure normality. We checked for linear relationships between independent and dependent variables and homoscedasticity by plotting standardized residuals as a function of standardized predicted values, which yielded no abnormalities. A Durbin‐Watson test and variance inflation factors indicated no problems with multicollinearity. Since the model does not violate the basic assumptions of multiple regression, we regard it as robust. The results of the OLS estimation are illustrated in Table 5.

**Table 5:** Results of the regression analysis

Variables	Hypothesis	Standardized coefficient (t statistic)
Regulation	H1	0.009 (0.143)
Internationalization	H2	0.034 (0.542)
C2C	H3	0.168* (2.339)
Management responsibility	H4a	0.250** (4.232)
Performance incentives	H4b	0.170* (2.210)
Employee awareness	H4c	0.122* (2.072)
Risk management	H5	0.236** (3.173)
Size		0.318** (3.927)
ROA		0.038 (0.573)
Leverage		−0.053 (−0.817)
Adjusted R^2^		0.680
F value		23.075**
Durbin‐Watson		2.302

*Note:* n = 105;

** p < 0.01;

* p < 0.05. C2C and ROA as given in the text.

Our model explains 68% of variation in the extent of corporate climate change action and the F value underlines the overall significance of coefficients. With regard to the research hypotheses, five out of seven can be accepted. Interestingly, the statistics suggest no relationship between *Climate action* and either *Regulation* (H1) or *Internationalization* (H2). *C2C,* however*,* is positively and significantly correlated with the extent of business responses to climate change (*β*_*3*_ *=* 0.168, *p* < 0.05), supporting theoretical assumptions that pressure is higher the closer a company operates to the end‐consumer market (H3). The three variables that measure the importance of organizational involvement for climate change action (i.e. *Management responsibility, Performance incentives*, and *Employee awareness*) all have a significant positive effect (*β*_*4*_ *=* 0.250, *p* < 0.01; *β*_*5*_ *=* 0.170, p < 0.05; *β*_*6*_ *=* 0.122, p < 0.05), allowing us to accept hypotheses H4a, H4b, and H4c. In line with H5, *Risk management* is another significant predictor (*β*_*7*_ *=* 0.236, p < 0.01). Concerning the control variables, only *Size* exercises a significant positive influence *(β*_*8*_ *=* 0.318, p < 0.01) on *Climate action*.

## Discussion

In light of constantly growing GHG emissions from the global transportation sector, it is crucial to understand the determinants of corporate responses to climate change in the automotive industry. By considering both the supply chain context and the global scale of the industry, the present study allows us to gain a more holistic understanding of institutional and intra‐organizational factors that trigger corporate climate action in the industry.

In terms of external factors, our paper contributes empirical knowledge to both institutional and stakeholder theories. The statistical analysis revealed that, in contrast to notions from institutional theory, regulatory pressures do not seem to be effective in facilitating the implementation of climate change strategies in the automotive industry. We could neither find a significant influence of a company's home country's climate policy regime nor its exposure to foreign institutional environments. The stringency of climate policies in a company's domestic market and the necessity to adapt to foreign regulations thus seem irrelevant for the implementation of appropriate response strategies. To examine the validity of this unexpected finding, we estimated alternative regression models that used proxies for the ambitiousness of environmental regulation other than the one considered in this study. We, inter alia, confronted our findings with results from models in which we substituted the CCPI with the Climate Laws, Institutions and Measures Index (European Bank for Reconstruction and Development, 2011) and the Environmental Sustainability Index (Esty *et al*., 2005). In each case, we could not detect any significant influences. This clearly contests common theoretical reasoning and results from other researchers suggesting that external political pressures are effective in making companies respond to climate change issues (Guenther *et al*., 2016; Luo and Tang, 2016). Having said this, our results should be viewed in line with their limitations. Since our analysis relies on cross‐sectional data, regulatory pressures might have unfolded their influential forces in the past and forfeited most of their efficacy by the time our investigation was undertaken. This might especially apply to the automotive industry because most industry‐related climate policies have existed for several decades and are often designed with a long‐term perspective in mind. Corporate average fuel economy standards in the US, for example, were introduced as early as 1975 (National Highway Traffic Safety Administration, 2017). Although these and similar standards are revised and tightened on a regular basis (ICCT, 2014), automotive companies might have already adapted their strategies to the respective regulatory environment before the years considered in our analysis.

In respect of stakeholder theory, our results highlight the vital role of pressure from primary stakeholders for driving business responses to climate change (Sprengel and Busch, 2011). We can confirm that a company's (*C2C*) is linked to the degree of action. This circumstance supports findings from Haddock‐Fraser and Tourelle (2010) and Guenther *et al*. (2016) who pointed out that consumers are an important stakeholder in the context of climate change. In accordance with the findings concerning other institutional pressures, we thus suppose that present corporate climate action in the automotive industry might rather be linked to reputational concerns than to compliance issues. On account of necessary interaction with end consumers, OEMs' activities are more ‘visible’ to the public than those of suppliers and therefore underlie increased public scrutiny. Consequently, demonstrating action on climate change might be a way of legitimizing a company's business operations. Yet, because our empirical analysis is based on secondary data, the higher level of engagement in climate change issues on the part of OEMs could also represent a form of ‘green washing’ rather than being a sign of actual action, which lies beyond the scope of our study.

The empirical results furthermore underline the importance of internal governance in the extent of companies' responses to climate change. Our paper empirically underpins ideas of the RBV in that more pronounced organizational capabilities can go hand in hand with taking climate change‐related measures (Backman *et al*., 2017). Moreover, the present study adds to the literature on the role of management characteristics, such as board structure, chief executive officers' educational background, or workforce diversity (Ciocirlan and Pettersson, 2012; Amran *et al*., 2016; Yunus *et al*., 2016). Specifically, we highlighted two additional important intra‐organizational drivers for corporate climate action. First, our analysis reveals the positive effect of involving the whole organization in climate change issues, including measures such as the assignment of responsibilities to top‐level managers, performance‐based remuneration schemes, and the creation of dedicated committees. In consequence, it seems to be crucial to establish clear leadership structures and to provide financial incentives to motivate action on climate change. Environmental education of employees forms another pillar of climate action as it seems to actuate pivotal organizational learning processes. Second, we discovered that companies which have integrated climate change in their risk management also exhibit a higher implementation level of response measures. Although research on the influence of distinct risk approaches is scarce, our findings support simulation results from Bleda and Shackley (2008), findings about managing the biophysical risks of climate change (Weinhofer and Busch, 2013), and studies that investigated the role of managers' perception of risk associated with pressure to reduce GHG emissions (Sangle, 2011; Boiral *et al*., 2012).

Our results are also in line with other management research that adduced company size to explain variation in climate change strategies (Lee S‐Y, 2012; Luo and Tang, 2016). As expected, larger firms show more extensive action on climate change, most likely because of easier availability of financial capital and human resources for implementation purposes. Larger companies additionally tend to be exposed to increased stakeholder scrutiny due to higher public visibility and media attraction (Bansal, 2005). We could not find proof, however, for an empirical relationship between the financial situation of a company and its climate change mitigation actions. This corroborates the mixed results of similar studies. While some authors have posited that the more economically successful a firm is the more likely it will pursue a sophisticated strategy (Bansal, 2005), others even discovered a negative linkage (Luo and Tang, 2016). The impact of financial performance thus remains ambiguous. The same holds true for the role of exposure to financial risks caused by a company's dependency on funding from creditors. Our results do not strengthen the conclusions derived by other scholars in that a higher degree of leverage leads to increased action on climate change (Delmas *et al*., 2016; Yunus *et al*., 2016). Instead, our investigation points at a rather negligible role of shareholder pressure which substantiates research on the determining factors of carbon disclosure (Guenther *et al*., 2016).

## Conclusions

The research objective of this paper was to better understand the causal mechanisms behind some internal and external factors for triggering action on climate change within the automotive industry. We found that intra‐organizational factors, such as organizational involvement and the integration of climate change into risk management, exhibit the greatest influence on corporate climate action. Our analysis additionally indicates that neither the stringency of a company's home country's climate policy regime nor the degree of internationalization is associated with a higher implementation level of response strategies. However, firms with business activities involving interaction with the end consumer tend to be more active, most likely because of their exposure to public scrutiny and customer pressure.

Our study has important implications for both managers and policymakers. First, it points out that owing to the absence of pressure from end consumers further upstream in the supply chain, automotive OEMs should exercise their buyer power to trigger action on climate change on the part of their suppliers. This is essential since OEMs increasingly outsource their manufacturing processes to suppliers and therefore, the effectiveness of OEMs' climate change strategies heavily depends on the performance of suppliers. Second, if companies want to be successful in embedding response strategies, they should be clear about the assignment of internal responsibilities for climate change and educate their workforce accordingly. Moreover, it seems to be beneficial to provide senior managers with financial incentives that are linked to a company's emission performance. Lastly, if consumer pressure is a major determinant of climate action, politicians might want to strategically strengthen the position of consumers in attempts to make companies act on climate change. One option might be to design policies aiming at changing consumer behaviour. In case vehicle emissions standards are not effective, subsidies for alternative fuel vehicles, for example, might create a shift in consumer demand and bring about changes in OEMs' product portfolios in favour of environmentally friendly propulsion systems.

Although our study extends knowledge about the determinants of corporate responses to climate change, it should be viewed in the context of its limitations. The use of voluntarily disclosed corporate information might limit the degree of reliability of the results obtained. Moreover, data quality might be impacted by inconsistencies in reporting practices or the lack of third‐party assurance. Another limitation concerns the generalizability of findings. As the focus was on globally leading automotive companies, the results might not apply to firms in other business sectors or to smaller companies within the same industry. In addition, Japanese firms made up a large share of our sample. Although this over‐representation of automotive companies from Japan is broadly in line with official industry statistics, it might have led to biased results.

We deem the following avenues particularly interesting for future research. First, subsequent studies might want to extend the analysis of climate action to small‐ and medium‐sized enterprises. Little is known about these types of companies, since data availability is a major constraint. Secondly, investigations that look at the effectiveness of sector‐specific legislation in triggering corporate climate action might yield important insights for policymakers. Third, since we found no evidence for a positive influence of regulatory pressures, it would be interesting to employ a longitudinal approach that investigates the relevance of climate policies for the evolution of business strategies over time.

## Appendix A

### Assessment of Climate Change Activities

**Table:**

GHG reduction policy	
Value	The company…
1	does not have a GHG inventory or reduction targets.
2	has estimated emissions but has no reduction targets.
3	has estimated its emissions and wants to reduce its emissions, without providing details.
4	has estimated its emissions, has reduction targets, and tracks emissions.

Product improvements	
Value	The company…
1	has not implemented product improvements.
2	has estimated product emissions without providing examples of product improvements.
3	provides examples of product improvements but no details about emission reductions.
4	provides examples of product improvements and details about emission reductions.

Process improvements	
Value	The company…
1	has not implemented process improvements.
2	has estimated process emissions without providing examples of process improvements.
3	provides examples of process improvements but no details about emission reductions.
4	provides examples of process improvements and details related to emission reductions.

New markets and products	
Value	The company…
1	does not plan to launch products or enter markets related to low‐carbon aspects.
2	has plans for launching products or entering markets.
3	prepares launch of products or market entrance.
4	has launched products or entered new markets.

Supplier involvement	
Value	The company…
1	has no plans to engage suppliers in climate change efforts.
2	has a general supply chain policy related to environmental protection without specific relation to climate change.
3	requests its suppliers to implement environmental management systems or to reduce emissions.
4	requests its suppliers to implement environmental management systems or to reduce emissions, and assists in implementing related measures.

Emission compensation	
Value	The company…
1	has not purchased emission credits via ETS or voluntary compensation schemes.
2	is preparing for ETS or voluntary compensation initiatives.
3	has acquired emission credits via ETS or is taking part in voluntary emission compensation initiatives.
4	has acquired emission credits via ETS and is taking part in voluntary emission compensation initiatives.

Stakeholder cooperation	
Value	The company…
1	does not plan to engage in stakeholder initiatives related to climate change.
2	has plans for engaging in stakeholder initiatives.
3	engages in one of the following:
	• stakeholder dialogues on climate change issues,
	• corporate citizenship initiatives related to climate change mitigation
4	engages in both above‐mentioned activities.

Carbon disclosure	
Value	The company…
1	reports about climate change aspects but does not have any formal reporting policy.
2	reports about climate change aspects and refers to reporting guidelines (e.g. CDP, GRI, ISO26000) without having a rating.
3	publicly reports about climate change aspects according to specific reporting guidelines and has a rating.
4	reports about climate change aspects according to specific reporting guidelines and ranks among the highest scoring bands (CDP score above 90, GRI A+).

Political lobbying	
Value	The company…
1	does not engage in political activities related to climate change.
2	engages in one of the following:
	• working together with trade associations or business initiatives,
	• funding of research organizations,
	• direct lobbying of policymakers.
3	engages in two of the above‐mentioned activities.
4	engages in all above‐mentioned activities.
